# Prevalence of neural epidermal growth factor-like 1- and exostosin 1/exostosin 2-associated membranous nephropathy: a single-center retrospective study in Japan

**DOI:** 10.1038/s41598-022-07037-2

**Published:** 2022-02-22

**Authors:** Takamasa Iwakura, Chiemi Ema, Shinsuke Isobe, Tomoyuki Fujikura, Naro Ohashi, Akihiko Kato, Hideo Yasuda

**Affiliations:** 1 Division of Nephrology, First Department of Internal Medicine, Hamamatsu University School of Medicine, 1-20-1 Handayama, Higashi-ku, Hamamatsu, 431-3192 Japan; 2 Blood Purification Unit, Hamamatsu University School of Medicine, Hamamatsu, Japan

**Keywords:** Epidemiology, Medical research, Nephrology

## Abstract

Membranous nephropathy (MN) is the leading cause of nephrotic syndrome in adults. We previously reported that the prevalence of phospholipase A2 receptor (PLA2R)- and thrombospondin type 1 domain containing 7A (THSD7A)-associated MN patients in Japan is 52.7% and 9.1%, respectively. In addition to PLA2R and THSD7A, we assessed the presence of newly discovered target antigens, neural epidermal growth factor-like 1 (NELL-1), semaphorin 3B (SEMA3B), and exostosin 1/exostosin 2 (Ext1/Ext2), in renal specimens from patients with primary and secondary MN by immunohistochemistry. We found enhanced glomerular staining of PLA2R, THSD7A, NELL-1, and Ext1/Ext2 in 53.6%, 8.7%, 1.5%, and 13.0% of the renal samples, respectively, in patients with primary MN. None of the patient specimens showed enhanced staining of SEMA3B. Enhanced glomerular staining of PLA2R, NELL-1, and Ext1/Ext2 was detected in 5.7%, 8.6%, and 22.9% of the patients with secondary MN, respectively. Based on our findings, we recommend the assessment of PLA2R, THSD7A and NELL-1 in addition to clinical information and IgG4 staining to differentiate between primary and secondary MN. This would aid in distinguishing secondary MN patients from primary MN patients who coincidentally have some secondary characteristics.

## Introduction

Membranous nephropathy (MN), caused by the formation of antigen–antibody immune complexes in the subepithelial region of the glomerular basement membrane (GBM), is the leading cause of nephrotic syndrome in adults^1^. MN is classified as primary or secondary MN. Hepatitis B and C viruses, autoimmune diseases, thyroiditis, malignancies, and the use of certain drugs are considered as secondary etiologies for MN^2^. MN without a secondary cause is termed primary MN; most of these cases present with serum autoantibodies against the glomerular podocyte antigens. Autoantibodies against the M-type phospholipase A2 receptor (PLA2R) and thrombospondin type-1 domain-containing 7A (THSD7A) are the primary causes of MN^3,4^. Autoantibodies against neural epidermal growth factor-like 1 protein (NELL-1) or semaphorin 3B (SEMA3B) are also thought to cause MN^5,6^. Alternatively, exostosin 1/exostosin 2 (Ext1/Ext2) have recently been identified as putative target antigens or glomerular markers in patients with secondary MN with autoimmune diseases^7^.

There are three ways to detect autoantibodies in the serum; western blotting, enzyme linked immunosorbent assay, and indirect immunofluorescence^8–17^. Renal immunohistochemical assessment of corresponding antigens identifies MN patients with autoantibodies, as the presence of serum autoantibodies against PLA2R/THSD7A/NELL-1/SEMA3B antigens enhance the antigens staining along GBM^5,12,14,16,18,19^. The combination of laser microdissection and tandem mass spectrometry has revealed increased glomerular protein levels of PLA2R, NELL-1, or SEMA3B in patients with the corresponding antibodies in their sera^5,6^.

The prevalence of PLA2R- and THSD7A-associated MN in the United States of America (USA) and Europe is approximately 70–80% and 1–5%, respectively^20,21^. The prevalence of NELL-1-associated MN is reported to be 16% in PLA2R/THSD7A-negative patients with MN^5^. The prevalence of SEMA3B-associated MN is unclear; however, these patients are predominantly diagnosed in childhood^6^. Accordingly, in USA and Europe, approximately 75–90% of the patients with primary MN show target antigens or autoantibodies. However, the same does not apply to Japanese patients; the prevalence of PLA2R- and THSD7A-associated MN in primary MN is reported to be approximately 33.6–50% and 3–9% in Japan, respectively^15,22–25^. Therefore, almost half of the Japanese patients with primary MN do not exhibit target antigens or autoantibodies.

We hypothesized that the prevalence of NELL-1- or SEMA3B-associated MN in Japan is higher than that in other countries. In addition, we investigated the prevalence and characteristics of Ext1/Ext2-associated patients in primary MN as well as secondary MN. We are extending our previous study^22^, and aim to demonstrate the detection rate of the target antigens in primary MN.

## Results

### Characteristics of primary and secondary MN

The number of patients assigned to the primary and secondary MN groups was 69 and 35, respectively. The secondary MN group comprised 14 patients with systemic lupus erythematosus and four patients with rheumatoid arthritis, three of whom were administered the anti-rheumatoid drug bucillamine. Six patients in this group had malignancies, including lung, colon, prostate, and uterine neoplasms; two were strongly suspected of having multiple myeloma or lymphoma. Five patients were infected with hepatitis B virus (HBV). Two patients were infected with hepatitis C virus (HCV). Two patients had thyroiditis. One patient was treated with nonsteroidal anti-inflammatory drugs. One patient had IgG4-related kidney disease. Among patients with lupus MN, six, three, and five patients were stratified under classes III + V, IV + V, and V, respectively.

**Figure 1:**
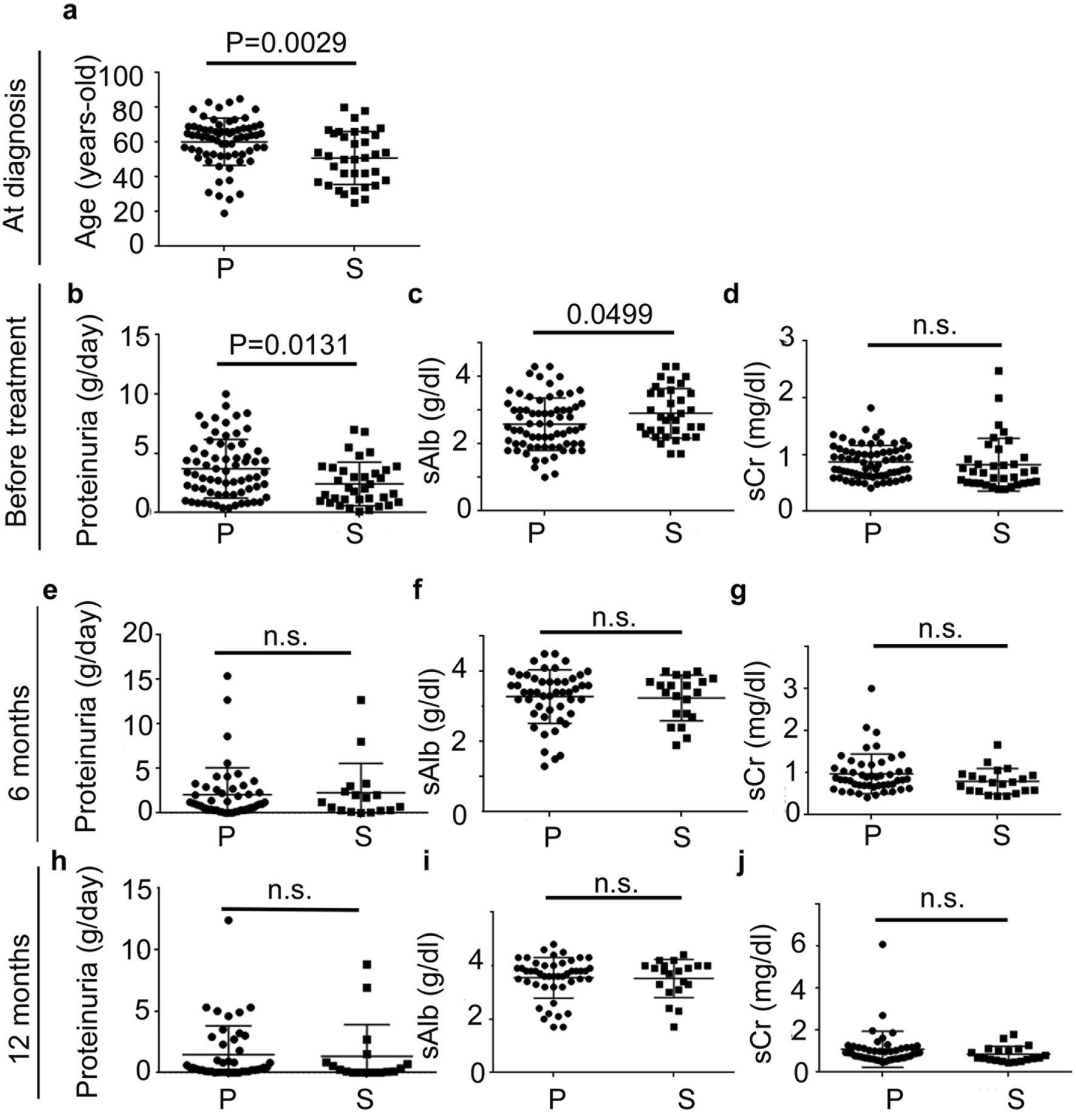
Changes in the clinical parameters of patients with primary and secondary MN during the 1-year follow up. (**a**) Age at diagnosis. The levels of proteinuria (**b**,**e**,**h**), serum albumin (**c**,**f**,**i**), and serum creatinine (**d**,**g**,**j**) just before the treatment (**b**,**c**,**d**), at 6 months (**e**,**f**,**g**), and at 12 months (**h**,**i**,**j**) after starting treatment. *P* primary membranous nephropathy, *S* secondary membranous nephropathy, *sAlb* serum albumin, *sCr* serum creatinine, *n.s*. not significance.

The characteristics of the patients with primary MN and secondary MN at the time of diagnosis, 6 months and 1 year after initiating treatment are summarized in Table 1 and Fig. 1. For secondary MN, the age of patients at diagnosis was lower than that for primary MN (p = 0.0029). The level of proteinuria was lower in the secondary MN group (p = 0.0131), whereas that of serum albumin was slightly higher (p = 0.0499) those in the primary MN group. Immunohistochemically, 59.4% of the samples from patients with primary MN were positive for IgG4; however, only 5.7% of the secondary MN samples were positive for IgG4 (p < 0.001). The percentage of patients followed up in our department over a year with primary and secondary MN was 71.0% and 48.6%, respectively. The complete or composite (complete or partial) remission rates at 6 months and 1 year were comparable in both primary and secondary MN groups. In the primary MN group, one patient was found to have esophageal cancer during follow up within 1 year. The patient’s sample showed moderate mesangial cell proliferation, which is one of the histological characteristics of cancer-associated MN^26^. However, the other samples from patients with malignancy did not show apparent mesangial cell proliferation. In the secondary MN groups, novel malignancies were not found during the follow up.

**Table 1:** Characteristics of primary and secondary MN patients at the time of renal biopsy and treatment response.

Characteristics	Primary	Secondary
Number of patients	69	35
Male; number (%)	40 (58.0)	16 (45.7)
Age, years; mean	60.2 ± 13.6	52.0 ± 15.8*
Proteinuria (g/day)	3.7 ± 2.5	2.5 ± 1.8^#1^*
Serum creatinine (mg/dl)	0.9 ± 0.3	0.8 ± 0.5
Serum albumin (g/dl)	2.6 ± 0.8	2.9 ± 0.7*
% of IgG4 positivity by IHC	59.4*	5.7
**Immunosuppressive therapy number (%)**		
Prednisolone	31 (44.9)	10 (28.6)
Prednisolone + Cyclosporine	13 (19.4)	0
Prednisolone + Azathioprine	1 (1.5)	1 (2.7)
Prednisolone + Cyclophosphamide	0	1 (2.7)
Prednisolone + Mizoribine	1 (1.5)	1 (2.7)
Prednisolone + Tacrolimus	0	1 (2.7)
Prednisolone + Mycophenolate mofetil	0	3 (8.1)
Total number (%)	46 (66.7)	17 (48.6)
**Clinical course**		
**6 months**		
Proteinuria (g/day)	1.9 ± 2.7	2.5 ± 3.3
Serum creatinine (mg/dl)	1.0 ± 0.5	0.8 ± 0.3
Serum albumin (g/dl)	3.3 ± 0.8	3.2 ± 0.7
% of complete remission (number)	24.5 (12)	33.3 (6)
% of composite remission (number)	57.1 (28)	61.1 (11)
**1 year**		
Proteinuria (g/day)	1.5 ± 2.4	1.6 ± 2.6
Serum creatinine (mg/dl)	1.1 ± 0.9	0.9 ± 0.4
Serum albumin (g/dl)	3.5 ± 0.8	3.5 ± 0.8
% of complete remission (number)	44.9 (22)	47.1 (8)
% of composite remission (number)	67.3 (33)	76.5 (13)

^#^1; a case did not check the level of proteinuria before renal biopsy. *p < 0.05.

### Prevalence of PLA2R-, THSD7A-, NELL-1-, SEMA3B- and Ext1/Ext2-associated primary and secondary MN

**Figure 2:**
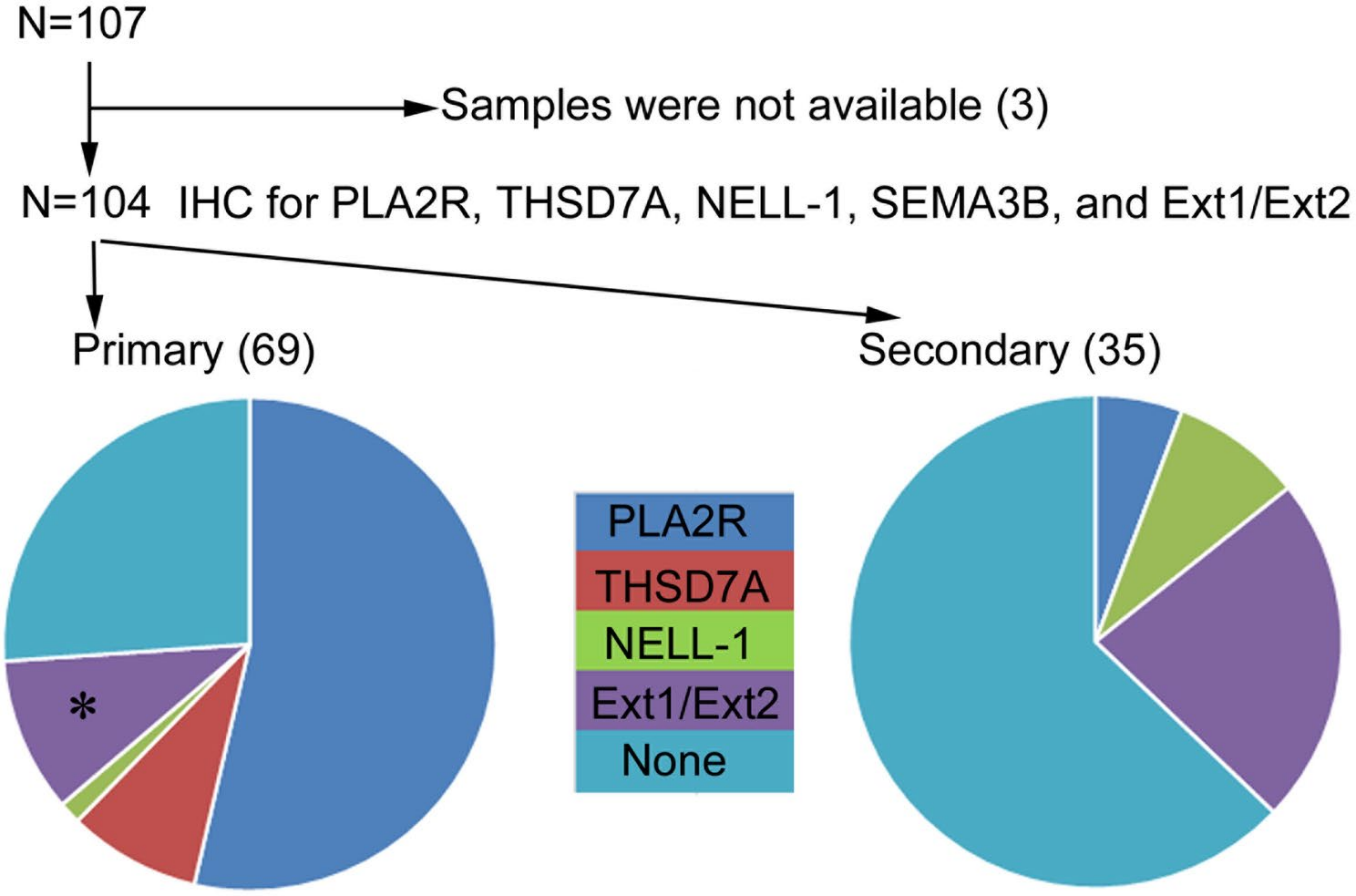
Flowchart of the patients’ classification. The percentage of each antigen in patients with primary and secondary MN is depicted in pie charts. *MN* membranous nephropathy, *IHC* immunohistochemistry, *PLA2R* phospholipase A2 receptor, *THSD7A* thrombospondin type-1 domain-containing 7A, *NELL-1* neural epidermal growth factor-like 1 protein, *SEMA3B* semaphorin 3B, *Ext* exostosin. *Two samples with enhanced staining of Ext1/Ext2 also had enhanced staining of other podocyte antigens (one sample was positive for PLA2R, and another was positive for THSD7A).

**Figure 3:**
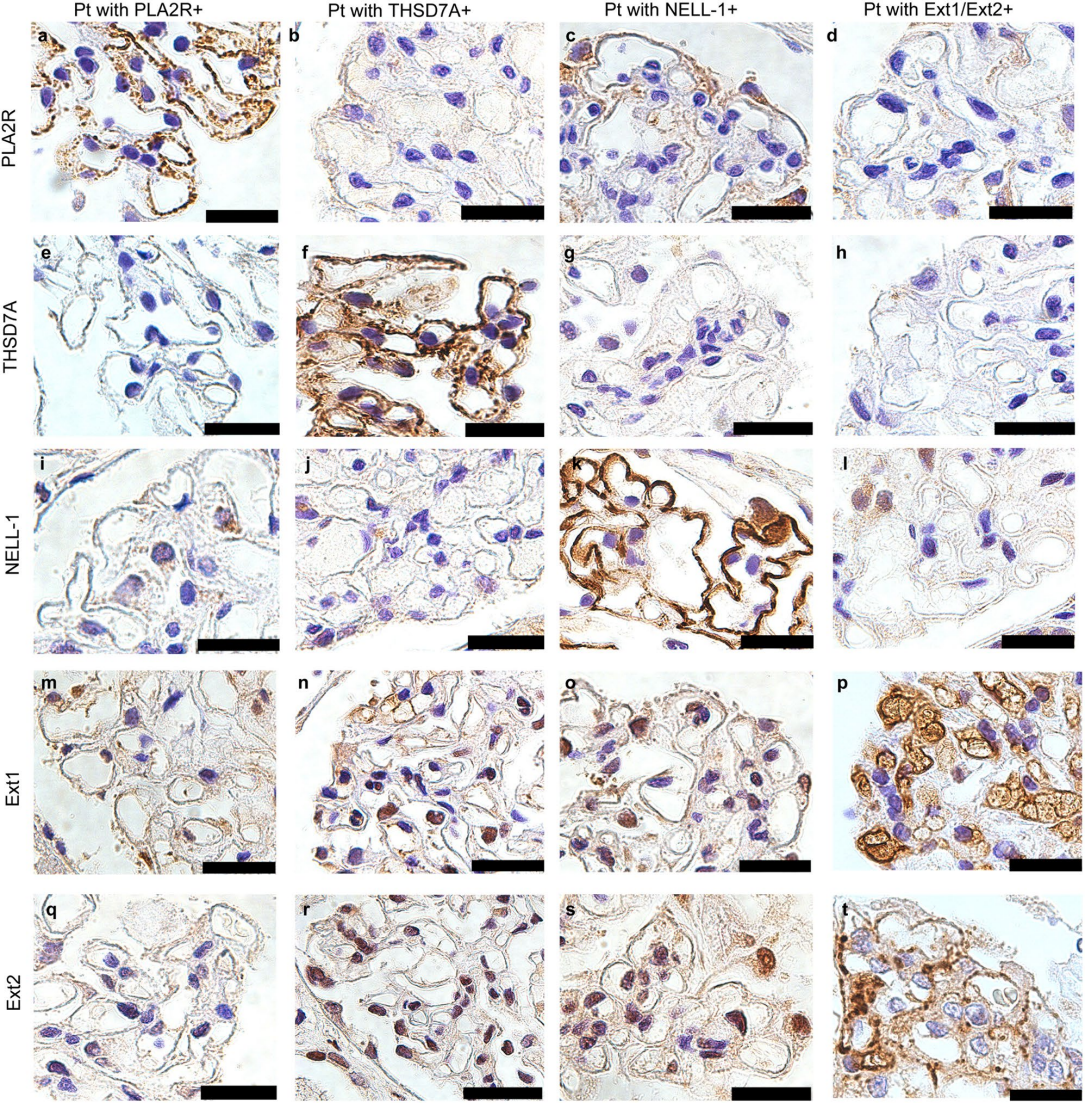
Immunohistochemical analysis for Phospholipase A2 receptor (PLA2R), Thrombospondin type-1 domain-containing 7A (THSD7A), Neural epidermal growth factor-like 1 protein (NELL-1), Exostosin (Ext) 1 and Ext2. Representative cases of immunostaining of PLA2R (**a**–**d**), THSD7A (**e**–**h**), NELL-1 (**i**–**l**), Ext1 (**m**–**p**) and Ext2 (**q**–**t**) in patients with primary membranous nephropathy. Original magnification: ×1000. Scar bar 25 μm. *Pt* patient.

**Figure 4:**
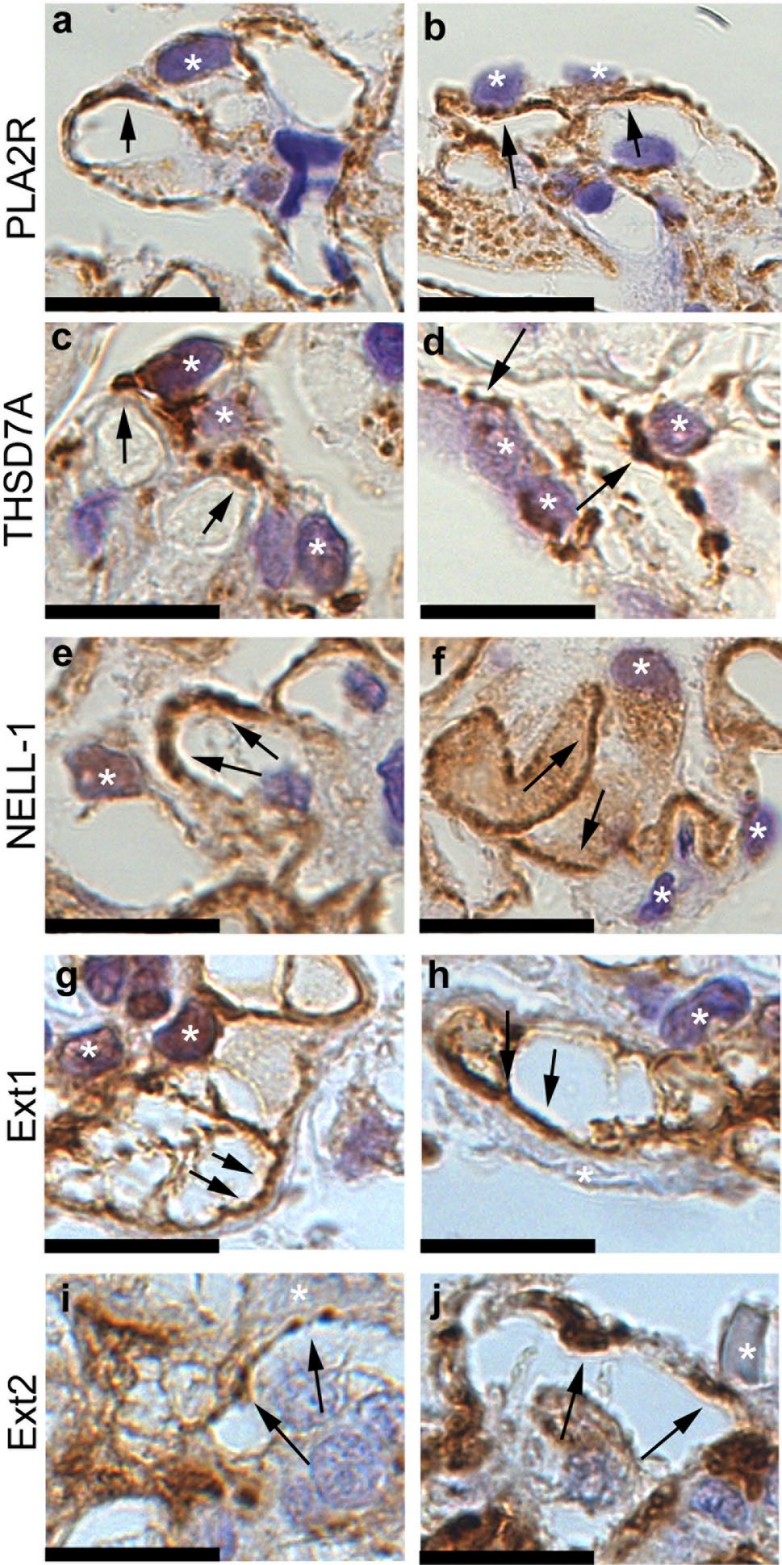
Immunohistochemical analysis for case of Phospholipase A2 receptor (PLA2R)-, Thrombospondin type-1 domain-containing 7A (THSD7A)-, Neural epidermal growth factor-like 1 protein (NELL-1)-, Exostosin (Ext) 1- and Ext2-associated membranous nephropathy with high magnification. Photographs of immunostaining of PLA2R (**a**,**b**), THSD7A (**c**,**d**), NELL-1 (**e**,**f**), Ext1(**g**,**h**) and Ext2 (**i**,**j**) with high magnification. The localization of enhanced antigens was shown with arrows. Asterisks; podocytes. Scar bar 12.5 μm.

Patient classification guidelines are shown in Fig. 2. Enhanced staining of PLA2R, THSD7A, NELL-1, and Ext1/Ext2 in the glomeruli was detected in 39 (37.5%), 6 (5.8%), 4 (3.8%), and 17 (16.3%) samples from the patients, respectively. None of the samples showed enhanced staining for SEMA3B. In the primary MN group, the prevalence of PLA2R-, THSD7A-, NELL-1, Ext1/Ext2-associated MN was 53.6%, 8.7%, 1.4% and 13.0%, respectively. Two patient samples with enhanced staining of PLA2R or THSD7A showed enhanced staining of Ext1/Ext2 as well. In the secondary MN group, 2 (5.7%), 3 (8.6%), and 8 (22.9%) patients had PLA2R-, NELL-1- and Ext1/Ext2-associated MN, respectively. Among patients with lupus MN, in three (60%) of the class V patients (n = 5), enhanced staining of Ext1/Ext2 was observed. Representative PLA2R-, THSD7A-, NELL-1, Ext1 and Ext2 staining in the glomeruli is shown in Figs. 3 and 4. Enhanced staining of each antigen was mainly along subepithelial and intramembrane (Fig. 4), suggesting that the localization of increased antigens was same to that of antigen–antibody immune complex deposits in MN.

### Characteristics of MN patients with enhanced staining of podocyte antigens

**Figure 5:**
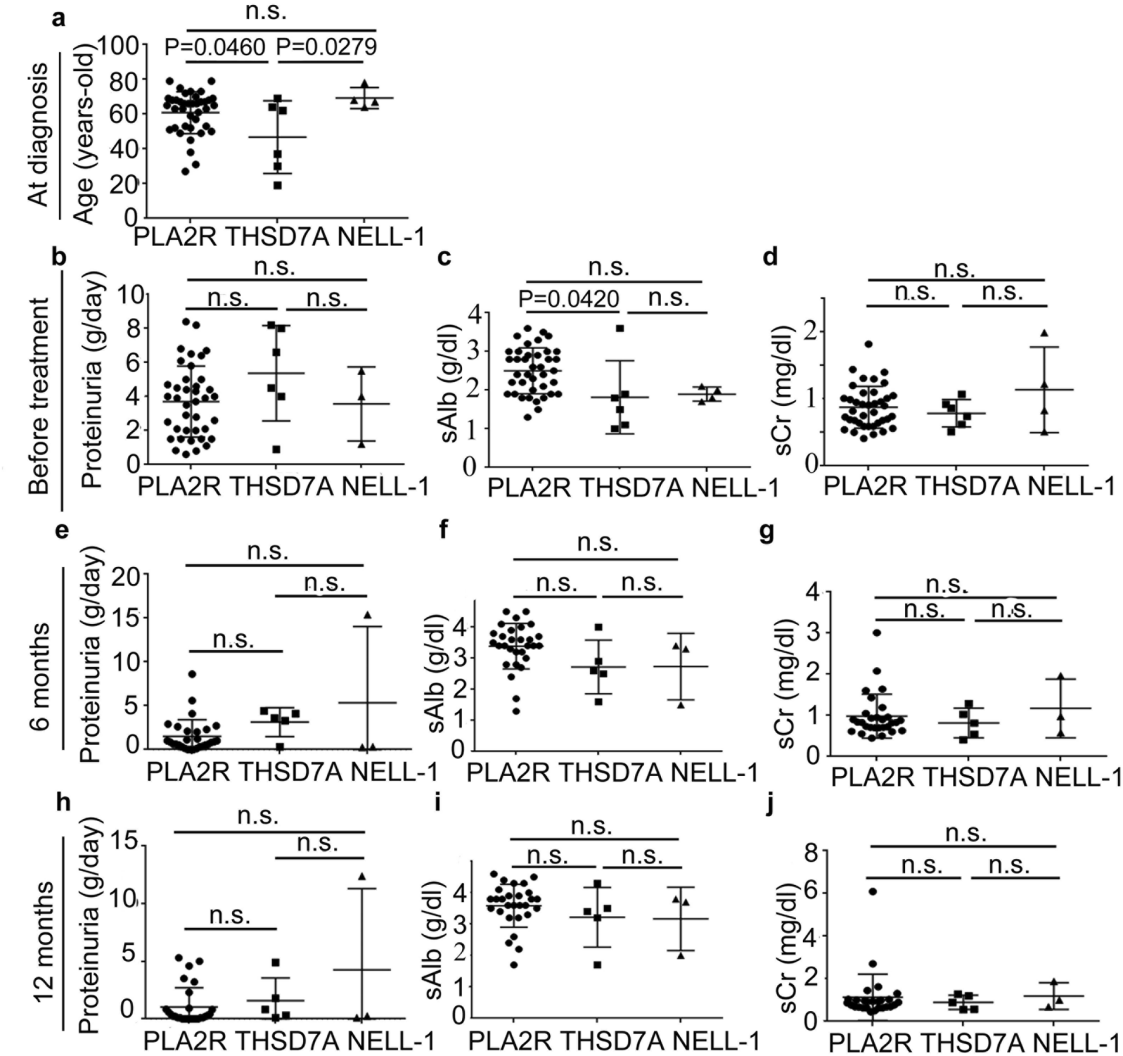
Changes in clinical parameters of each podocyte antigen-associated MN patients during the 1-year follow up. (**a**) Age at diagnosis. The levels of proteinuria (**b**,**e**,**h**), serum albumin (**c**,**f**,**i**), and serum creatinine (**d**,**g**,**j**) just before the treatment (**b**,**c**,**d**), at 6 months (**e**,**f**,**g**), and at 12 months (**h**,**i**,**j**) after starting treatment. *sAlb* serum albumin, *sCr* serum creatinine, *n.s*. not significance.

The clinical characteristics of the patients associated with each target antigen at the time of renal biopsy, 6 months and 1-year post-treatment are summarized in Table 2 and Fig. 5. As autoantibodies against Ext1/Ext2 have not discovered yet^7^, the result of Ext1/Ext2 was separated in other section. The age at diagnosis for THSD7A-associated MN was younger than those for PLA2R- and NELL-1-associated MN (p = 0.0460 or 0.0279). Immunosuppressive therapy was initiated in 69.2% (27 of 39 cases), 83.3% (5 of 6 cases), and 50.0% (2 of 4 cases) of the patients with PLA2R-, THSD7A-, and NELL-1-associated MN, respectively. The level of serum albumin at baseline was lower for THSD7A-associated MN, compared with those for PLA2R-associated MN (p = 0.0420). However, the difference disappeared by 6 months. There were no differences between NELL-1-associated and PLA2R-associated MN. No patients from the THSD7A- and NELL-1-associated MN groups experienced malignancy. Immunohistochemically, 92.3%, 66.7%, and 25% of the samples from patients with PLA2R-, THSD7A-, and NELL-1-associated MN were positive for IgG4 in glomeruli. The rate of IgG4 positivity in the glomeruli was significantly higher in PLA2R-associated MN, compared with NELL-1-associated MN (p = 0.0061).

**Table 2:** Characteristics of each podocyte antigen-associated MN patients at the time of renal biopsy and treatment response.

Characteristics	PLA2R	THSD7A	NELL-1
Number of patients	39	6	4
Male; number (%)	25 (64.1)	2 (33.3)	4 (100)
Age, years; mean	60.9 ± 12.1	46.8 ± 20.8	69.2 ± 6.1
Proteinuria (g/day)	3.7 ± 2.1	5.4 ± 2.8	3.6 ± 2.2 ^#1^
Serum creatinine (mg/dl)	0.9 ± 0.3	0.8 ± 0.2	1.1 ± 0.6
Serum albumin (g/dl)	2.5 ± 0.6	1.8 ± 0.9*	1.9 ± 0.2
Cancer (previous/present)	3 (2/1)	0	0
Autoimmune disease	0	0	2
% of IgG4 positivity by IHC	92.3*	66.7	25
**Immunosuppressive therapy number (%)**			
Prednisolone	15 (38.4)	3 (50.0)	1 (25)
Prednisolone + Cyclosporine	11 (28.2)	2 (33.3)	0
Prednisolone + Azathioprine	1 (2.6)	0	0
Prednisolone + Cyclophosphamide	0	0	1 (25)
Prednisolone + Tacrolimus	0	0	0
Total number (%)	27 (69.2)	5 (83.3)	2 (50.0)
**Clinical course**^**#2**^			
**6 months**			
Proteinuria (g/day)	1.9 ± 2.8	3.0 ± 1.9	5.3 ± 8.7
Serum creatinine (mg/dl)	1.0 ± 0.5	0.8 ± 0.4	1.2 ± 0.7
Serum albumin (g/dl)	3.4 ± 0.7	2.8 ± 1.0	2.7 ± 1.1
% of complete remission (number)	24.1 (7)	20.0 (1)	66.7 (2)
% of composite remission (number)	72.4 (21)	60.0 (3)	66.7 (2)
**1 year**			
Proteinuria (g/day)	1.3 ± 2.0	1.5 ± 2.3	4.3 ± 7.1
Serum creatinine (mg/dl)	1.1 ± 1.1	0.8 ± 0.3	1.2 ± 0.6
Serum albumin (g/dl)	3.6 ± 0.7	3.2 ± 1.1	3.2 ± 1.0
% of complete remission (number)	55.2 (16)	40.0 (2)	66.7 (2)
% of composite remission (number)	79.3 (23)	80.0 (4)	66.7 (2)

^#^1; a case did not check the level of proteinuria before renal biopsy. #2; 10, 1 and 1 patients' data in PLA2R-, THSD7A- and NELL-1-associated MN patients were not available because of follow-up in other hospitals. *p < 0.05.

In the PLA2R-associated MN group, two patients had a previous history of cancer, and one patient was diagnosed with cancer during the screening for secondary MN causes. However, the patients achieved remission without cancer treatment. A patient who was a hepatitis B carrier achieved spontaneous remission.

We found that all four patients with NELL-1-associated MN were male. Detailed patient characteristics are shown in Table 3. Three patients were clinically diagnosed with secondary MN; two with rheumatoid arthritis and one was administered nonsteroidal anti-inflammatory drugs. A patient was considered to have primary MN due to positive staining of IgG4 in the glomeruli. Glomerular IgG4 staining was negative for the other patients.

**Table 3:** Clinical information in each case of NELL-1–associated MN.

Case	Age	Sex	Proteinuria (g/day)	Serum creatinine (mg/dl)	Serum albumin (g/dl)	Complications	Clinical diagnosis	Immunosuppressive therapy	Time to remission
1	68	Male	1.2	0.51	2.0	Rheumatoid arthritis	Secondary MN (autoimmune disease)	Prednisolone	< 6 months
2	67	Male	Not available	0.83	1.7	Rheumatoid arthritis	Secondary MN (anti-rheumatoid drug)	Prednisolone + Cyclophosphamide	< 6 months
3	78	Male	5.5	1.99	2.1	DM	Secondary MN (NSAIDs-induced)	None	NA
4	64	Male	4	1.22	1.8	DM, ANA, anti-ssDNA	Primary MN (IgG4+)	None	Not yet; 12 months pass

*DM* diabetes mellitus, *ANA* antinuclear antibody, *ssDNA* *Ab* single strand DNA antibody, *MN* membranous nephropathy, *NSAIDs* nonsteroidal anti-inflammatory drugs, *NA* not available.

### Characteristics of patients with Ext1/Ext2-associated MN

A total of 17 patients had enhanced staining of Ext1/Ext2 in the glomeruli. Detailed patient characteristics and the comparison between the primary and secondary MN groups with the Ext1/Ext2-associated MN are shown in Tables 4 and 5, respectively. There were 11 male (64.7%) and six female (35.2%) patients. Among these patients, nine and eight patients with primary and secondary MN, respectively, presented enhanced Ext1/Ext2 glomerular staining. In patients with lupus MN, enhanced Ext1/Ext2 staining was observed in three (60%) of the class V patients (n = 5). None of the samples from the lupus class III + V (n = 6) or class IV + V (n = 3) showed enhanced staining. The mean age at presentation was 51.4 ± 13.3. The mean serum creatinine, serum albumin, and proteinuria levels at diagnosis were 0.8 ± 0.3 mg/dl, 3.0 ± 0.9 g/dl, and 2.5 ± 2.3 g/day, respectively. The rate of IgG4 positivity in the glomeruli was 13.3% (2 of 15) when excluding two patients with other podocyte antigens (#8 and #9). This was lower than those observed for PLA2R- and THSD7A-associated MN (p < 0.0001 or 0.0307, respectively).

**Table 4:** Clinical information in each case of Ext1/Ext2–associated MN.

Case	Clinical diagnosis	Age	Sex	Proteinuria (g/day)	Serum creatinine (mg/dl)	Serum albumin (g/dl)	Complications	ANA/dsDNA/others	C3/C4/CH50	IHC of IgG4	Immunosuppressive therapy
1	Primary	55	Female	0.8	0.53	2.9	None	neg/NA/NA	Normal	neg	None
2	Primary	63	Male	2.1	1.00	3.2	None	neg/NA/NA	Normal	pos	Prednisolone + Cyclosporine
3	Primary	62	Female	1.3	0.48	2.5	None	neg/NA/NA	Normal	neg	Prednisolone
4	Primary	59	Male	2.5	1.16	4.3	None	neg/NA/NA	Normal	neg	None
5	Primary	53	Male	1.1	1.35	4.0	None	NA/NA/NA	NA	neg	Prednisolone
6	Primary	49	Male	0.6	0.73	3.8	None	320/neg/neg	Normal	neg	None
7	Primary	29	Female	6.1	0.51	2.0	None	320/neg/neg	Normal	pos	Prednisolone
8	Primary ^#1^	49	Male	2.1	0.68	3.0	None	neg/neg/neg	Normal	pos	Prednisolone
9	Primary^#2^	69	Female	8.2	1.07	1.5	None	neg/NA/NA	Normal	pos	Prednisolone + Cyclosporine
10	Secondary	25	Male	0.2	0.70	4.3	Hepatitis B	neg/NA/NA	Normal	neg	None
11	Secondary	37	Male	1.5	0.68	2.2	Hepatitis B	neg/NA/NA	Normal	neg	Interferon
12	Secondary	65	Male	6.7	0.95	1.7	Prostate cancer Ulcerative colitis Guillain–Barre syndrome	neg/neg/neg	Normal	neg	Prednisolone
13	Secondary	35	Female	3.3	0.58	2.2	Thyroiditis	1280/neg/neg	Normal	neg	Prednisolone
14	Secondary	67	Male	2.1	0.83	3.5	Lung cancer	neg/NA/NA	Normal	neg	None
15	Secondary	46	Male	1.2	0.80	3.5	Lupus	320/neg/RNP, ssDNA,SS-A	33/9/ < 10	neg	Prednisolone + Tacrolimus
16	Secondary	59	Female	1.8	0.72	2.7	Lupus + scleroderma	640/neg/ssDNA	34/14/5	neg	Prednisolone + Mycophenolate mofetil
17	Secondary	52	Male	0.5	0.50	4.0	Lupus	1280/neg/ssDNA	Normal	neg	Prednisolone

*ANA* antinuclear antibody, *neg* negative, *pos* positive, *NA* not available, *ssDNA* single strand DNA, *dsDNA* double strand DNA, *#1* enhanced staining of PLA2R was also found, *#2* enhanced staining of THSD7A was also found.

**Table 5:** Characteristics of Ext1/Ext2-associated MN in primary and secondary MN patients at the time of renal biopsy and treatment response.

Characteristics	Total	Primary	Secondary
Number of patients	17	9	8
Male; number (%)	11 (64.7)	5 (55.6)	6 (75.0)
Age, years; mean	51.4 ± 13.3	54.2 ± 11.6	48.3 ± 15.2
Proteinuria (g/day)	2.5 ± 2.3	2.8 ± 2.6	2.2 ± 2.1
Serum creatinine (mg/dl)	0.8 ± 0.3	0.8 ± 0.3	0.7 ± 0.1
Serum albumin (g/dl)	3.0 ± 0.9	3.0 ± 0.9	3.0 ± 0.9
**Immunosuppressive therapy number (%)**			
Prednisolone	7 (41.2)	4 (44.4)	3 (37.5)
Prednisolone + Cyclosporine	2 (11.8)	2 (22.2)	0
Prednisolone + Tacrolimus	1 (5.9)	0	1 (12.5)
Prednisolone + Mycophenolate mofetil	1 (5.9)	0	1 (12.5)
**Clinical course**^**#1**^			
**6 months**			
Proteinuria (g/day)	1.7 ± 1.2	1.8 ± 1.2	1.5 ± 1.3
Serum creatinine (mg/dl)	0.9 ± 0.3	0.9 ± 0.3	0.8 ± 0.1
Serum albumin (g/dl)	3.4 ± 0.5	3.4 ± 0.5	3.5 ± 0.6
% of complete remission (number)	22.2 (2)	16.7 (1)	33.3 (1)
% of remission (number)	66.7 (6)	66.7 (4)	66.7 (2)
**1 year**			
Proteinuria (g/day)	1.4 ± 1.7	1.7 ± 2.1	0.7 ± 0.8
Serum creatinine (mg/dl)	1.0 ± 0.4	1.1 ± 0.5	0.8 ± 0.3
Serum albumin (g/dl)	3.9 ± 0.4	3.9 ± 0.5	4.0 ± 0.2
% of complete remission (number)	44.4 (4)	50.0 (3)	33.3 (1)
% of remission (number)	66.7 (6)	66.7 (4)	66.7 (2)

^#^1; Follow up was done in our department in 6 and 3 patients of primary and secondary MN group, respectively.

**Figure 6:**
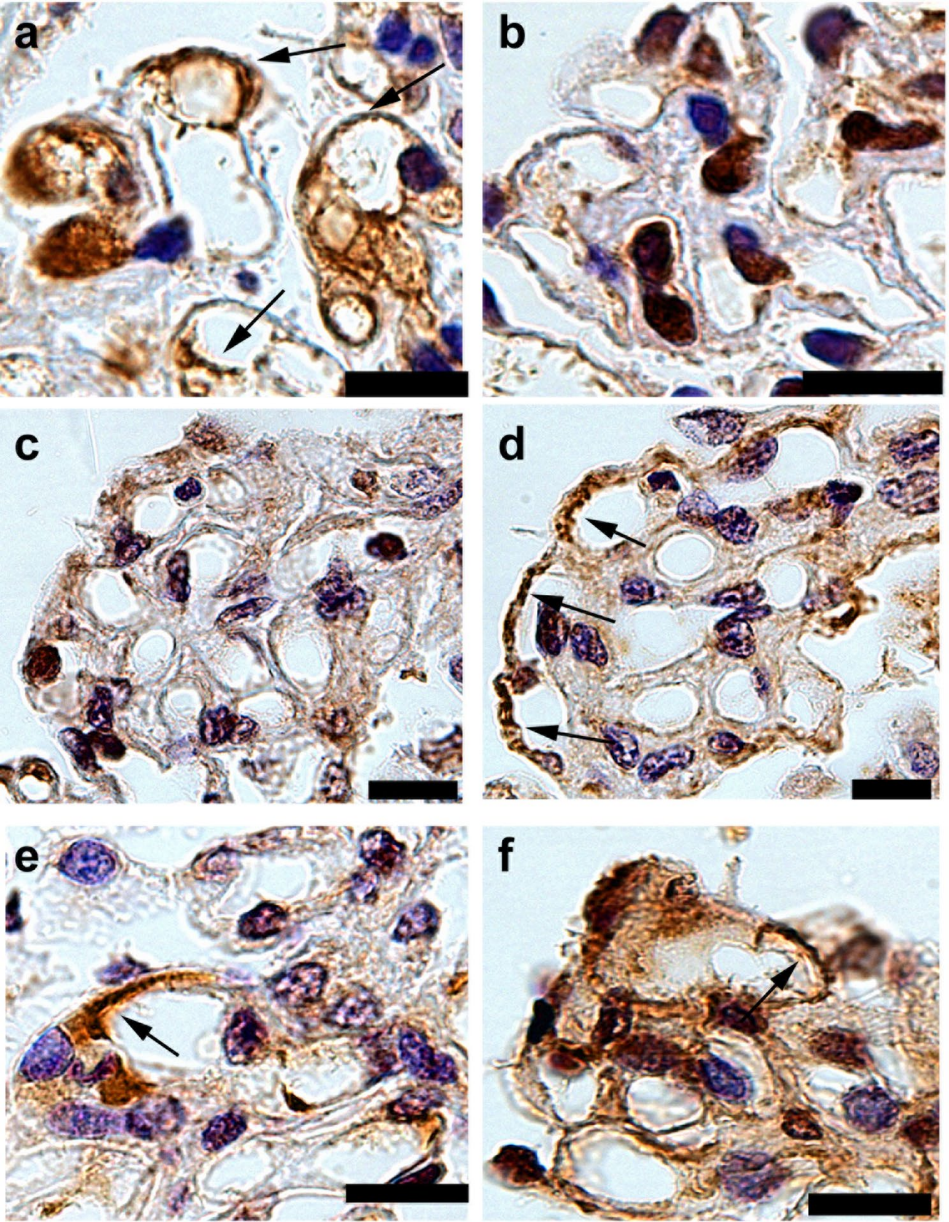
Immunohistochemical analysis of exostosin (Ext) 1 and Ext2 in the atypical cases of Ext1/Ext2-associated membranous nephropathy (MN). Photographs of immunostaining of Ext1 (**a**,**c**,**e**) and Ext2 (**b**,**d**,**f**). A sample in primary MN showed positive staining of Ext1 (**a**), but negative staining of Ext2 (**b**). In lupus MN patients, a sample showed negative staining of Ext1 (**c**), but segmental, positive staining of Ext2 (**d**), while another sample showed segmental, positive staining of Ext1 (**e**) and Ext2 (**f**). Original magnification: ×1000. Scar bar 12.5 μm.

There were no significant differences between the primary and secondary MN groups. In the primary MN group, antinuclear antibody (ANA) was present in two patients. None of the patients showed any symptoms of autoimmune diseases at the time of the renal biopsy. One patient subsequently exhibited a transient decrease in the lymphocyte number; however, the patient did not satisfy the criteria for the diagnosis of lupus. Four patients had positive glomerular IgG4 staining, whereas two others had enhanced staining of other podocyte antigens (#8; PLA2R, #9; THSD7A). For one patient from the primary MN group, enhanced Ext1 staining and negative Ext2 staining (Fig. 6a,b) were observed. In the secondary MN group, ANA was present in four patients. Cancer was found in two patients (prostate and lung carcinoma); two had an HBV infection, and one had thyroiditis at the time of diagnosis. None of the patients showed positive glomerular IgG4 staining. Among patients with lupus MN, two showed segmental and enhanced Ext2 staining (Fig. 6d,f). The Ext1 staining was either negative or scarce (Fig. 6c,e). All patients in the lupus MN were treated with immunosuppressive therapy. However, these patients were not followed-up in our department, and the data of urine were not available. Meanwhile, immunosuppressive therapy was administered in 57.1% (8 of 14 cases) of the non-lupus patients, which was equivalent rate with other forms of MN. Of them, nine were followed up until 1 year after treatment. The complete/composite remission rates in 6 months and 1 year were 22.2%/66.7% and 44.4%/66.7%, respectively.

## Discussion

Our findings revealed that the prevalence of enhanced PLA2R, THSD7A, NELL-1, and Ext1/Ext2 staining in the glomeruli detected by immunohistochemical analysis of biopsy samples was 53.6%, 8.7%, 1.5%, and 13.0%, respectively, in the studied cohort of Japanese patients with primary MN. We could not detect any samples showing enhanced staining of SEMA3B, which probably because our cohort only included adult patients. Therefore, approximately 80% of the patients with primary MN were detected using the target antigens, recognized through immunohistochemical analyses. That is, the remaining 20% of the patients may have unidentified autoantibodies in their sera or an unrecognized secondary cause of MN. In consistent with our previous work^22^, the prevalence of PLA2R-associated MN was lower than that in other countries^8,9,11–14,16,17^, and equivalent with the rates reported from Japan^15,23^. Alternatively, the prevalence of THSD7A-associated MN was higher than that reported in other studies^19,24^. These differences might be due to genetic and/or geographical differences between reports^5,23,24^.

The prevalence of NELL-1-associated MN in PLA2R/THSD7A-negative, non-lupus MN accounted for 8.9% (4 of 45 cases) of the cases, with this percentage being lower than that reported by Sethi et al.^5^ and higher than that reported by others^27,28^. The association of cancer with NELL-1 is a major concern^5^, as demonstrated in THSD7A-associated MN^29,30^. However, no cancers were detected in all our patients with NELL-1-associated MN. Given that our study only included a small number of patients with NELL-1-associated MN, it was impossible to exclude the concern regarding the association of NELL-1 and cancer, thus further investigation is required. Of note, 3 of 4 NELL-1-associated MN patients were clinically diagnosed with secondary MN because of the existence of a secondary cause for MN and negative staining for IgG4 in the glomeruli. As IgG4 is part of the predominant IgG subclass against PLA2R and THSD7A autoantibodies, patients with positive IgG4 staining in the glomeruli were previously thought to have primary MN. However, IgG4 staining can no longer determine primary or secondary MN as IgG1 is also a part of the predominant IgG subclass against NELL-1 and SEMA3B autoantibodies^5,6^. Therefore, we conclude that positive staining for IgG4 is indicative of either PLA2R/THSD7A-associated MN or IgG4-predominant and unidentified autoantibody-associated MN, but not necessarily primary MN.

The prevalence of Ext1/Ext2-associated MN was 26.7% (12 of 45 cases) in PLA2R/THSD7A-negative, non-lupus MN. Sethi et al. reported that 70.8% of the patients with Ext1/Ext2-associated MN had abnormal autoimmune laboratory findings, and 84.6% of the renal specimens from these patients showed features suggestive of a secondary MN related to an autoimmune disease. This demonstrated that Ext1/Ext2 represent potential biomarkers or target antigens in secondary autoimmune MN^7^. In our study, 60% of the samples (3 of 5 cases) from patients with lupus class V showed positive staining of Ext1/Ext2, supporting that they are associated with autoimmune diseases. However, it is of note that ANA was present in only 21.4% of the patients (3 of 14 cases), and all patients with Ext1/Ext2-associated non-lupus MN had normal complement levels. Furthermore, patients with Ext1/Ext2-associated MN in our study were predominantly male and older than the cohort described in the report from Sethi et al.^7^. However, these patients might be diagnosed with autoimmune diseases in the future. Our results suggested that Ext1/Ext2 could occur without the presence of autoimmune diseases. Interestingly, our study included two patients with Ext1/Ext2-associated MN that showed positive staining for other podocyte antigens^31^. Further case accumulation is needed to clarify the roles and features of Ext1/Ext2-associated MN.

In our study, two patients with PLA2R-associated MN were assigned to the secondary MN group by conventional classification based on clinical information. These patients achieved remission without treatment for diseases associated with the secondary cause of MN, suggesting that these patients developed PLA2R-associated MN and had stochastically developed diseases associated with the secondary causes of MN. These cases emphasize that the existence of a secondary cause of MN does not exclude enhanced expression of podocyte antigens or existence of autoantibodies against podocyte antigens in the sera. Additionally, the existence of autoantibodies against podocyte antigens does not eliminate a secondary cause of MN. Meanwhile, a patient in the primary MN group developed cancer during the follow up within 1 year. The patient’s sample showed moderate mesangial cell proliferation, suggesting that the patient had cancer-associated secondary MN. However, only this case had apparent mesangial cell proliferation in MN patients with malignancy, suggesting that absence of mesangial cell proliferation cannot exclude the existence of malignancy. It has been reported that malignancy was discovered in only 20% of the patients before the diagnosis of MN^32^ and was detected within 1 year of the diagnosis of MN in 80% of the patients^33^. However, a high risk of cancer persists for at least 5 years^33^. Therefore, we propose immunohistochemical examination for podocyte antigens and/or the detection of the serum levels of anti-podocyte antibodies in all patients with MN, in addition to the conventional classification based on clinical information, and recommend careful observation for more than 5 years. This strategy could prevent patients with primary MN from missing out on effective immunosuppressive therapies, and might decrease the likelihood of misdiagnosing diseases with secondary cause of MN (especially malignancy) in anti-podocyte antibody-positive MN patients.

As our cohort was retrospectively studied for 25 years, some patients were not treated with the latest standard treatment regimen recommended by the international guidelines (Kidney Disease: Improving Global Outcomes, KDIGO^34^. For example, steroid monotherapy was the most opted treatment in our cohort, but it is not recommended for MN in the KDIGO guidelines^35^. On the contrary, steroids have been used as first-line drugs for the initial treatment of nephrotic patients with primary MN in Japan, and they have been associated with a favorable prognosis^36^. The study of Shinki et al. involved a large cohort of 949 Japanese nephrotic patients with primary MN. Steroid monotherapy was the most opted treatment (39.4%), followed by cyclophosphamide combined with steroids (27.1%) and other immunosuppressive agents such as cyclosporin, mizoribine, and azathioprine (16.5%). The complete or composite [complete + incomplete (defined as proteinuria less than 3.5 g/day)] remission rate during follow-up (6.4 ± 4.3 years) in nephrotic patients with primary MN treated with steroid monotherapy was approximately 50% or 90%, respectively, and the 15-year renal survival rate in these patients exceeded 80%. In addition, the renal survival rate between patients treated with steroid monotherapy and those treated with cyclophosphamide combined with steroids was not significantly different^36^. Based on available evidence and concerns of toxicity, the use of cyclophosphamide is avoided in Japan. However, recently, we have been modifying the treatment regimen in accordance with the KDIGO guidelines, and primary MN patients with a moderate risk have been treated with cyclosporin and steroids. Regarding rituximab, the KDIGO guidelines recommend its use for MN patients with a moderate or high risk^34^, but its use has not been covered by health insurance as an initial treatment for MN patients in Japan.

This study has several limitations. Firstly, this single-center study included a single geographical region with a small sample size. Therefore, the prevalence might differ from that in Japan as a whole. Second, PLA2R-, THSD7A-, NELL-1-, SEMA3B-, and Ext1/Ext2-associated MN were examined only by immunohistochemistry. Therefore, patients with only autoantibodies in the serum might be missed. Autoantibody testing in blood should be a part of future studies in order to better estimate the prevalence of new antigens. Third, we could not exclude the possibility that our immunohistochemical analysis for SEMA3B was challenging due to the absence of a positive control sample in our experiment. Finally, the treatment regimens in this cohort differed from that recommended by KDIGO^34^. Therefore, it is not possible to compare this cohort to cohorts in other countries in terms of treatment outcome.

In conclusion, we determined that the prevalence of enhanced glomerular staining of NELL-1 or Ext1/Ext2 in Japanese patients with PLA2R/THSD7A-negative, non-lupus MN was 8.9% and 26.7%, respectively. Enhanced glomerular staining of NELL-1 and Ext1/Ext2 was detected in 8.6% and 22.9% of the patients with secondary MN, respectively. The combined prevalence of patients with primary MN, showing enhanced staining of PLA2R, THSD7A, NELL-1, and Ext1/Ext2 was approximately 80%. Meanwhile, 14.3% of the patients in the secondary MN group were positive for podocyte antigens (5.7% for PLA2R, 8.6% for NELL-1). We recommend the inclusion of the immunohistochemical evaluation of NELL-1, PLA2R, and THSD7A, in addition to IgG4 staining, for an improved classification of primary and secondary MN. Our results demonstrate the necessity to change the classification of MN from an etiologic point of view to a podocyte antigen/antibody-associated disease in the future.

## Materials and methods

### Patients and classification

This study examined 107 consecutive adult patients (age > 18 years; 57 men and 50 women) with the histological diagnosis of MN established in our institution from January 1995 to January 2020. We collected the clinical information and laboratory data at the time of biopsy, 6 months and 1 year after initiating treatment through a review of patient medical records. All patients underwent rigorous screening for the secondary causes of MN, which included serological analysis, physical examination, obtaining information on prescribed medications, and testing for malignancies. MN subjects with diseases associated with secondary MN were classified as having secondary MN^22^. The diagnosis of systemic lupus erythematosus was made based on the American College of Rheumatology revised criteria for the classification of systemic lupus erythematosus in 1997^37,38^. Samples from three patients were unavailable due to a lack of glomerular samples for evaluation. The remaining patients were divided into primary MN and secondary MN groups, based on clinical data. The mean number of glomeruli in the samples of these patients was 21.4 ± 9.5, and 92.3% of the samples included a sufficient number of glomeruli (at least 10 glomeruli)^39^. The mean number of glomeruli with global sclerosis was 2.4 ± 2.8. Grouping was conducted by a well-trained nephrologist (C.E.) with blinded histological findings. Patients with lupus nephritis were assigned to the lupus MN group. Patients who were found to have a secondary cause of MN by screening were assigned to the secondary MN group. Patients who had been treated for cancer previously, but completed treatment for malignancy and had not had recurrence within 1 year of MN diagnosis were assigned to the primary MN group. The remaining patients were also assigned to the primary MN group.

Immunohistochemistry for PLA2R, THSD7A, NELL-1, SEMA3B, Ext1, Ext2, and IgG4 was performed in all cases. Disease severity was assessed based on levels of proteinuria, serum albumin, and serum creatinine at the time of biopsy. Complete remission was defined as proteinuria of < 0.3 g per 24 h and a serum albumin level of > 3.5 g/dL. Partial remission of proteinuria was defined as proteinuria < 3.5 g/24 h and at least a 50% reduction from the time of inclusion^40^. The study protocol was approved by the research ethics committee of the Hamamatsu University School of Medicine (#E20-165). All research was conducted in accordance with the ethical principles stated by the Declaration of Helsinki. The requirement for obtaining informed consent was waived by the research ethics committee based on the retrospective design of this study. A detailed disclosure of the study contents was published on the website of the research ethics committee. Patient records/information was anonymized and de-identified prior to analysis. Fully anonymized data of patients' medical records were accessed from July 2020 to December 2020. We selected the patients who sought treatment from January 1995 to January 2020 and collected data from their whole medical records.

### Histology

We examined paraffin-embedded tissue sections derived from patients with MN. Immunohistochemistry for PLA2R, THSD7A, and IgG4 was performed as described previously^22^, and that for NELL-1, SEMA3B, and Ext1/Ext2 were carried out as described by Sethi et al. with slight modifications^5–7^. Briefly, 3 μm paraffin-embedded sections of renal biopsy specimens from patients with MN were deparaffinized and rehydrated. Antigen retrieval was achieved by boiling in Histofine Antigen Retrieval Solution pH9^®^ (Nichirei Bioscience, Tokyo, Japan) or citrate buffer solution (Mitsubishi Chemical Medience Corporation, Tokyo, Japan) for 20 min at 100 °C. Nonspecific binding was blocked with Protein Block Serum-Free^®^ (#X0909; DAKO, Glostrup, Denmark) for 15 min at room temperature prior to incubation with anti-PLA2R (1:8000, #HPA012657; Atlas Antibodies, Stockholm, Sweden), anti-THSD7A (1:400, #HPA000923; Atlas Antibodies), anti-IgG4 (1:500, #HP6025, Binding Site, CA, USA), anti-NELL-1 (1:100, #PA5-106907; Thermo Fisher Scientific, Waltham, MA, USA), anti-SEMA3B (1:200, #ab48197; Abcam plc, Cambridge, UK), anti-Ext1 (1:100, #HPA012657; Atlas Antibodies), and anti-Ext2 (1:100, #ab102843, Abcam plc) antibodies in phosphate-buffered saline supplemented with 1% bovine serum albumin. The primary antibodies were reacted with Histofine Simple Stain MAX PO^®^ (Nichirei Bioscience) and were visualized using a standard peroxidase-diaminobenzidine system. Nuclei were counterstained with hematoxylin. Positive staining was defined as the presence of dark brown granular deposits (i.e., enhanced staining) whose boundary was clear on the GBM. Histological classification of lupus nephritis was determined by two nephrologists (T.I. and C.E.) independently after anonymizing clinical and histological information.

### Statistical analysis

All values are expressed as the mean ± standard deviation. Differences in categorical outcomes were evaluated using either the chi square test or the Fisher’s exact test, as appropriate. Differences between the two groups were assessed using Mann–Whitney U test. Differences between three or more groups were examined using analysis of variance, followed by Tukey’s post hoc test. All statistical analyses were performed using GraphPad Prism version 6 (GraphPad Software, San Diego, CA, USA). Statistical significance was set at p < 0.05.
